# Systematic approach for dissecting promoters and designing transform systems in microalgae

**DOI:** 10.1186/s12934-025-02700-5

**Published:** 2025-05-29

**Authors:** Qinhua Gan, Xinjun Yu, Xiang Jiang, Xingwei Huang, Yi Xin, Yandu Lu

**Affiliations:** 1 State Key Laboratory of Marine Resource Utilization in South China Sea, School of Marine Biology and Fisheries, Hainan University, Haikou, 570228 China; 2 Hainan Engineering & Research Center of Marine Bioactives and Bioproducts, Haikou, 570228 China; 3 Haikou Innovation Platform for Research & Utilization of Algal Bioresource, Hainan University, Haikou, China; 4 School of Tropical Crops, Hainan University, Haikou, 570228 China

**Keywords:** Gene regulation, Synthetic biology, Oleaginous microalgae, *Nannochloropsis*

## Abstract

The rapidly expanding catalog of sequenced genomes has revolutionized the pace and scale of microalgal cellular metabolism delineation. However, knowledge of the gene regulation in these genomes is lacking. This is true even for *Chlamydomonas reinhardtii*, the laboratory model species where transcriptional regulation is best understood, although systematic knowledge of regulatory elements (e.g., promoters) remains elusive. This leads to limitations in microalgae for engineering system designs, which currently rely mainly on characterizing the molecular mechanisms of individual regulatory sequences via low-throughput methods. Here, we take a first step toward multi-promoter dissection and demonstrate systematic dissection of multiple microalgal promoters through quantitative genome-wide comparisons and sequence-based structural annotations. We demonstrate this approach on both well-studied and previously uncharacterized promoters in the oleaginous microalga *Nannochloropsis oceanica* IMET1. Using in silico design, representative regulatory elements were synthesized and assembled as building blocks for transgene circuits. Assessment of the in vivo activity revealed the maximum transformation efficiency (414 ± 102 transformants/µg DNA) in the plasmid pHN5-2 containing *lhp* promoter and *α-tub* terminator. Therefore, the present synthetic approach for establishing engineering systems is superior to the conventional empirical methods. Applying these transforming circuits to the creation and characterization of a gene-indexed loss-of-function mutagenesis library verifies the applicability of this strategy to the discovery and standardization of regulatory parts for microalgae. Moreover, the generality of the approach presented here provides the possibility of quantitative promoter dissection and rational transform system design in *N. oceanica* and a wide range of other microalgae.

**Supplementary Information:**

The online version contains supplementary material available at 10.1186/s12934-025-02700-5.

Microalgae exhibit significant potential as feedstock for CO_2_ sequestration [1] as well as sustainable and scalable production of valuable molecules ranging from therapeutic proteins to biofuels [2–4]. However, few natural strains exhibit the traits required for feedstock associated with value-added chemical production. This lack has spurred the search for more genomic characterization and biological technologies (than those currently available) for microalga-based investigations [5]. Molecular technologies have considerable potential for accelerating the process of conventional genetic breeding. Although the field of microalgal genetic engineering has advanced significantly over the past decade [6, 7], routine transformation has only been achieved in a few algal species [8–10].

Rational methods of establishing competent transformation systems are lacking, thereby representing a major limitation for most microalgae being developed for high-value chemical production [11, 12]. The ability to transform (using vector constructs) algal cells with DNA sequences of interest constitutes a crucial component of transgenic-microalgae creation. However, there are limited options with the algal vectors currently available, owing partly to the paucity and inefficiency of the genetics toolbox (i.e., regulatory elements) available for these organisms. Most studies are performed empirically with a relatively small number of repurposed parts and without predictive modeling. Moreover, the lack of reasonable strategies for the development of reverse or forward genetic engineering technologies further hampers rational trait-improvement [8, 13].

Promoters represent critical elements that work in concert with other regulatory regions (enhancers, silencers, boundary elements/insulators, etc.) to direct the transcription of a given gene. Although many genomic and transcriptomic studies have been performed in microalgae, a thorough understanding of the diversity and complexity characterizing the regulatory mechanism of microalgae remains elusive [14]. Furthermore, discovery and characterization of regulatory elements (e.g., promoters) is indispensable for the design of artificial biological systems and understanding the natural counterparts of such systems.

*Nannochloropsis* spp. have emerged as a model for functional genomics, owing to relatively small genome sizes (~ 30 Mb), high-quality sequences, and extensive functional genomics resources [15–22]. Moreover, they are considered to be a potential feedstock for fuels and high-value products due to rapid growth, broad environmental tolerance, as well as large accumulations of value-added chemicals (e.g., eicosapentaenoic acid or EPA). Due to these distinct advantages, the list of sequenced *Nannochloropsis* genomes has increased significantly [15, 16, 18–20]. These genome sequences along with omics technologies (*i*) facilitate systematic investigations of various cellular mechanisms [17, 21, 23–31] and (*ii*) serve as an ideal catalyst for the development and application of integrated and extensible strategies via the characterization, standardization, and use of an inventory of basic biological parts as building blocks for transgene expression [32].

Employing the industrial oleaginous microalga *Nannochloropsis oceanica* strain IMET1 as a model, we demonstrated a rational, combinatorial, and genomic approach for establishing engineering systems. Temporal tracking of transcripts at a genome scale revealed a comprehensive quantitative picture of the dynamics associated with promoter strengths. The promoters, featured modules, and terminators were assembled as a serial of transgene circuits, which were characterized in vivo in terms of transformtion efficiency for IMET1. These circuits were then successfully used to create an insertional mutagenesis library with a high efficiency and a random distribution of insertion sites in the genome. The combination of bottom-up engineering mindset (including DNA construction, parts libraries, computational design, as well as manipulation and probing of synthetic circuits) are important steps enabling the full of genomic data comprising oleaginous microalgae.

## Results

### Genome-scale identification of promoters for establishing scalable libraries

For improved understanding of regulatory elements, we began by performing mRNA-Seq. This mRNA-Seq generated numerous expressed sequence tags, thereby allowing the statistical comparison of the expression level characterizing each gene and exploration of the gene structure, expression, and regulation information. To maximize the coverage of transcripts sampled, the transcriptomic dynamics of IMET1 was monitored over a full-time course (initial OD_750_ is 2.0, then culturing during 3, 4, 6, 12, 24, or 48 h, Supplemental Dataset 1) [23].

The transcript abundance (TA) of each gene at a particular time point was determined based on the Fragments Per Kilobase of exon model per Million mapped fragments (FPKM) value. A total of 9756 genes, accounting for 91.7% of all the annotated genes in the IMET1 genome [15, 23], were aligned with at least one read FPKM value (≥ 1) for each biological sample (Figure S1A). An average FPKM value of 61 was obtained for all the genes at six time points (Figure S1B), and the TA of each gene was sorted according to the FPKM values at these points. Genes with FPKM values among the top 1000 at any of these points were nominated as TOP 1000 (Supplemental Dataset 1). An average FPKM value of 319 was obtained for these 1000 genes, which were then grouped into 10 clusters based on their temporal expression patterns (MATERIALS AND METHODS; Figure S1A and Supplementary Dataset 1). To determine if a given expression pattern is linked to specific biological functions, the annotated genes in each cluster were categorized into 11 functional groups via Kyoto Encyclopedia of Genes and Genomes (KEGG) (MATERIALS AND METHODS; Figure S1C and Supplemental Dataset 1). The genes with unknown functions account for 22–77% of the genes in each cluster, representing the largest category. In contrast, protein metabolism (which accounted for 63% of all genes) is the predominant category in cluster 4. For the clusters consisting predominantly of unknown genes, the second largest functional category is designated as the primary functional genes (Figure S1C). The primary functional genes of seven clusters are involved in metabolism.

Among the TOP 1000 genes, those with FPKM values > 319 at all six time points have (in general) high-strength constitutive promoters, and were nominated as TOP 100 (124 genes; Supplemental Dataset 2). The average FPKM value of the TOP 100 genes is 1108, and 38% of these genes are related to protein metabolism and folding. Furthermore, most of the TOP 100 genes encode ribosomal proteins, indicative of protein synthesis during cell proliferation. Enrichment of the photosynthesis category may have also contributed to the robust growth of IMET1. Therefore, by grouping above genes based on their temporal expression patterns, clustering helps distinguish promoters that drive consistent and high expression levels for further promoter recovery.

**Fig. 1:**
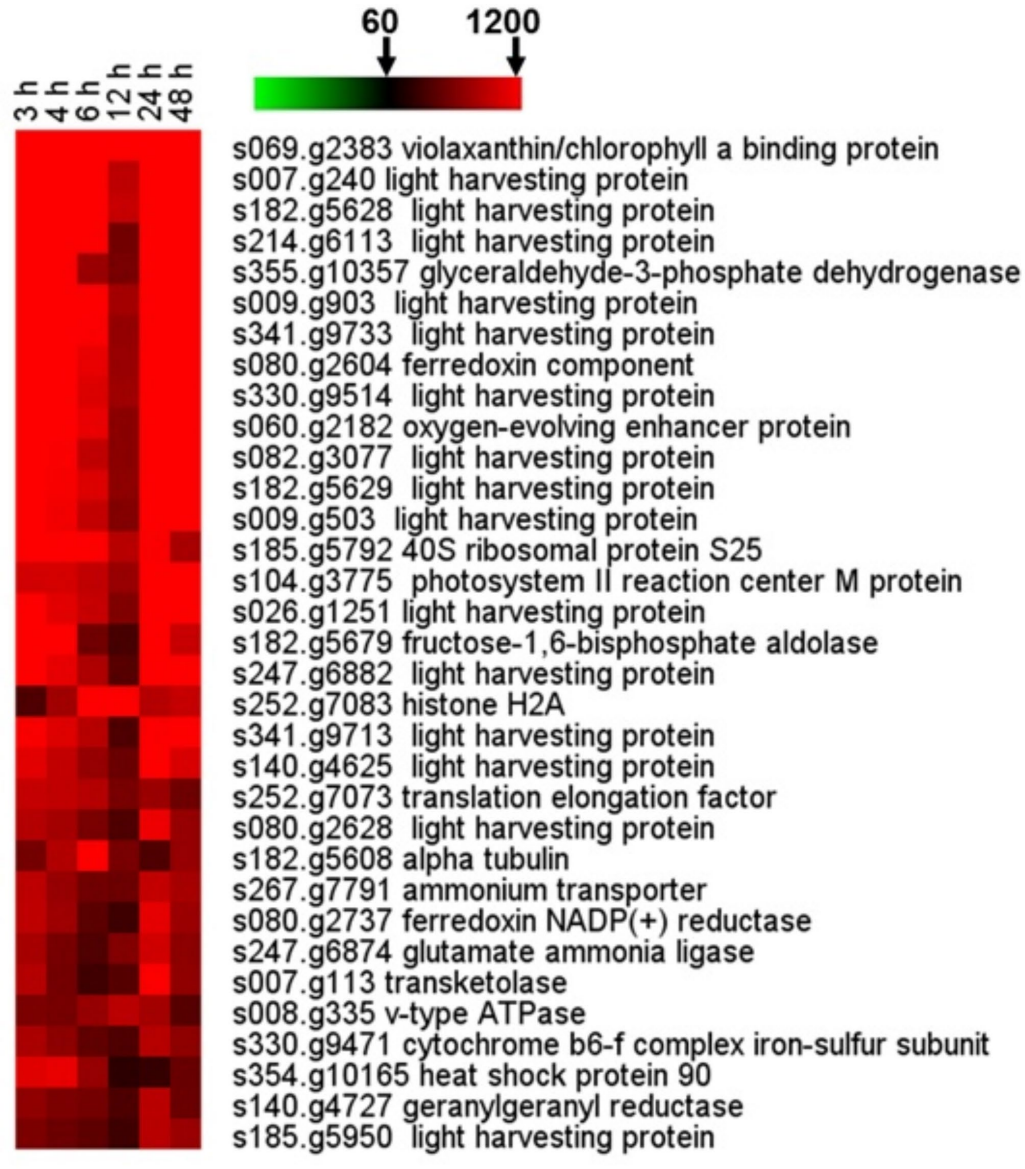
Heatmap of FPKM value of the top 30 genes in the TOP 100. The average FPKM value of all genes (61) is set as the threshold. Red areas indicate transcripts with FPKM values of > 1200

### Recovery of well-characterized promoters through genome-wide comparison

Stable expression over time-course is crucial for constitutive promoter selection. Thus, a heatmap was generated at 3 h to 48 h after inoculation, displaying the FPKM values of the top 30 differentially expressed genes among the TOP 100 genes (Fig. 1 and Supplementary Dataset 2). The threshold for inclusion was set at the average FPKM value (61) of all genes. The heatmap reveals that a significant proportion of the genes exhibit high expression levels, particularly at earlier time points (3 h, 4 h and 6 h). Among the top 30 genes, 18 genes are related to photosynthetic apparatuses, such as light harvesting complexes and photosystem reaction center proteins, such as s007.g240, s182.g5628, s214.g6113, s341.g9733, etc., suggesting a strong induction of photosynthesis-related pathways. Other highly expressed genes include those encoding glyceraldehyde-3-phosphate dehydrogenase (GAPDH), ferredoxin component, oxygen-evolving enhancer protein, and photosystem II reaction center M protein, which are crucial for carbon fixation and electron transport. The expression of genes gradually declined after 12 h, with most transcript levels dropping at 48 h. The results revealed that all top 30 promoters display stability over time-course under *N. oceanica* culturing.

To demonstrate that mRNA-Seq acts as a tool for discovering regulatory elements *de novo*, we searched for the (*i*) well-characterized promoters in *Nannochloropsis* or (*ii*) promoters where the homologs in closely-related microalgal species are routinely employed for driving constitutive gene expression. Violaxanthin/chlorophyll (a binding protein (*VCP*, s069.g2383) [33]) and the elongation factor (EF, s252.g7073) [34], which have been routinely applied to *Nannochloropsis* transformation, were found in the TOP 100 genes (Supplementary Dataset 2). Owing to their high constitutive activity, promoters of *tubulin *and heat shock protein (*HSP*) have been studied extensively and used to drive constitutive gene expression in the nucleus of *Chlamydomonas* and several other microalgae [19, 35, 36]. *HSP70A* (g5260) and alpha tubulin (s182.g5608) are also included in the TOP 100 genes (Fig. 1 and Supplementary Dataset 2). Therefore, this genome-wide view of the transcriptome provides a viable method of searching for promoters with strong activities that drive the foreign gene expression in microalgae.

### Discovery of regulatory architectures in unannotated promoters via genome-wide comparison

**Fig. 2:**
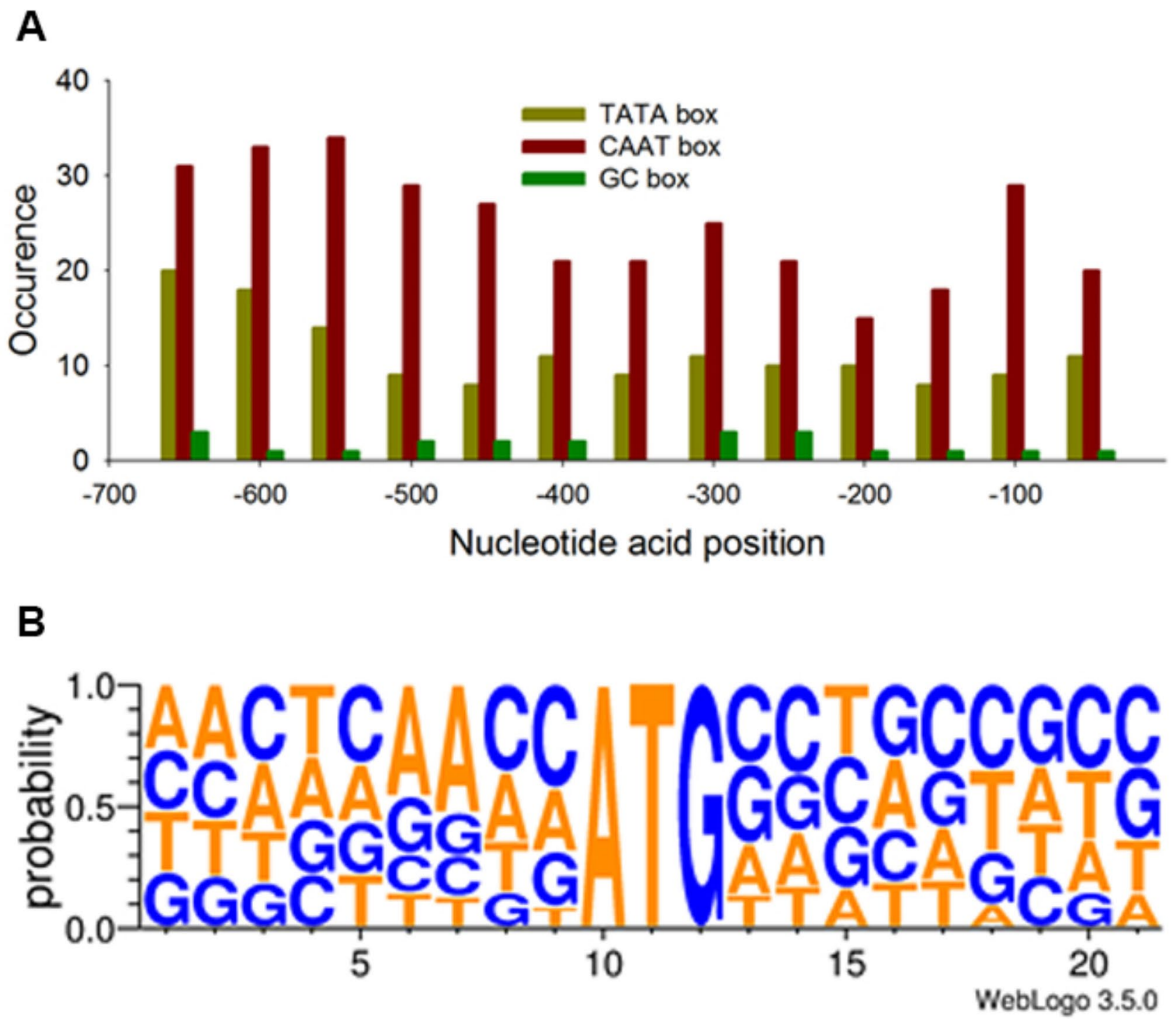
Occurrence of featured modules in promoter regions of TOP 100 genes. (**A**) Occurrence and location in the promoter regions of TOP 100 genes associated with the featured modules, including the TATA box (sequence TATAAA), CAAT box (sequence GGCCAATCT), GC box (GGGCGG), and OCT element (ATTTGCAT). (**B**) Occurrence frequency of the nucleotide acid around the initiation codon from the mRNAs of trimmed TOP 100 genes. The height of each stack and the height of the nucleotides represent the consensus sequences at that position (in bits) and the relative frequency of each nucleotide at that position, respectively

The identification of regulatory architectures via this genome approach was then demonstrated. For this identification, several featured modules in the promoter are necessary for the binding of an RNA polymerase II. For example, the TATA box (sequence TATAAA), CAAT box (sequence GGCCAATCT), GC box (GGGCGG), and OCT element (ATTTGCAT) are core elements in many eukaryotic promoters [37]. The promoter region is typically divided into three parts: the (*i*) core promoter (i.e., the region responsible for the actual binding of the transcription apparatus), which is typically located − 35 base pairs (bp) upstream of the transcription start site (TSS), (*ii*) proximal promoter, a region containing several regulatory elements, which lies up to a few hundred bp upstream of the TSS, and (*iii*) distal promoter, which can lie several thousands of bp upstream of the TSS and contains additional regulatory elements referred to as enhancers and silencers. We focused our investigation on the core promoter regions. The upstream regions 700 bp from the TSS of the TOP 100 genes were retrieved. We determined the occurrence and location of the four elements in each gene (Fig. 2A and Supplementary Dataset 3). For several genes in the TOP 100, all four elements are absent in upstream sequence, possibly due to incorrect annotation or bidirectional promotion (further identifications are ongoing). Thus, to ensure availability, promoters containing some or all of the aforementioned elements were utilized to construct a refined TOP 100 library.

A translation process where the initiation is determined by the Kozak consensus sequence in eukaryotes occurred in the second layer for transgene expression [38]. The A nucleotide of the AUG triplet was referred to as number + 1, while the A at the − 3 and a purine at + 4 (preferably G) being the most and second-most influential, respectively. To determine the genome-wide presence or absence of the Kozak consensus sequence in IMET1, initiation codons were first annotated for the trimmed TOP 100 genes. The frequency of nucleotide acid occurrence was calculated for positions ranging from − 9 to 12 (Supplementary Dataset 4). In IMET1, these sites are basically matched to the Kozak consensus sequence (Fig. 2B). High frequencies of purine occurrence (G: 32.8%, C: 32.8%) are determined for position + 4, while A occurred mainly (52.9%) at position − 3. Subsequently, we created an in silico characterization by a library of constitutive promoters with strong activities. The creation of this library (based on a constitutive promoter) would eliminate the need for regulating inducer concentrations and avoiding heterogeneities in cellular response [39]. However, owing to the complexity of the biological systems, prediction of promoter functionalization is difficult. Therefore, we further explored the in vivo activities of selected promoters.

### Rational design of transgene circuits using standardized regulatory parts

**Fig. 3:**
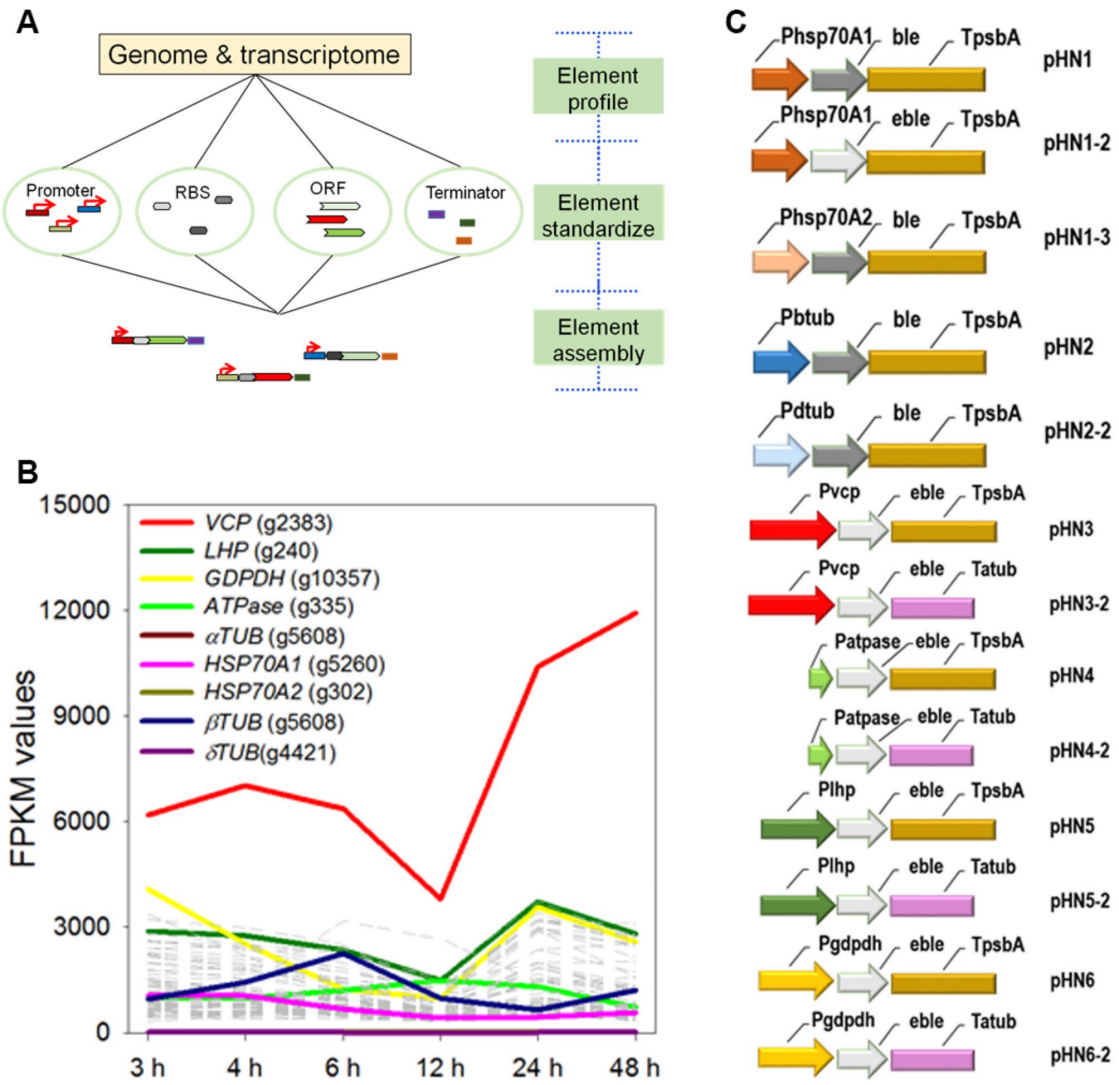
Customized design and assembly of transforming circuits. (**A**) Schemetic of expression circuit assemblies. (**B**) The transcript abundance of genes where the promoter or terminator regions were used for expression circuit assembly. The grey and dotted lines indicate the transcriptional dynamics of the TOP 1000 genes. FPKM: Fragments Per Kilobase of exon model per Million mapped fragments. (**C**) Transforming circuits devised in this study. Abbreviation: VCP: violaxanthin/chlorophyll, a binding protein (s069.g2383), ATPase: v-type ATPase (s008.g335), LHC: Light harvesting complex protein (s007.g240), GDPGH: glyceraldehyde-3-phosphate dehydrogenase (s355.g10357), FD: ferredoxin component (s080.g2604), psbA: chloroplast gene, photosystem II protein D1 (s001.27891-29028), aTUB: alpha tubulin (s182.g5608), *BLE*: zeocin resistance gene, *eBLE*: codon-optimized *BLE*, HSP70A: heat shock protein 70 A (s162.g5260)

Characterization and standardization of regulatory parts (e.g., promoters, coding sequences, and terminators) are essential for the design and assembly of efficient expression circuits (Fig. 3A). Here, the transcriptional level and the expression level were considered. Transcriptional initiation, where promoters were retrieved from the trimmed TOP 100 promoter library, and mRNA stabilization were both probed at the transcriptional level. To include a broad range of characteristics on regulatory elements, three functional categories were considered (see Fig. 3B and Table S1 for the details of each category) namely, the: (*i*) photosynthetic genes, specifically the genes encoding the light harvesting complex protein (*LHC*, s007.g240; average FPKM value: 2672) and the well-characterized *Nannochloropsis* constitutive promoter of the *VCP* gene were selected, (*ii*) energy-related genes, the gene encoding v-type ATPase (*ATPASE*, s008.g335) with an average FPKM value of 1118 was recruited, and (*iii*) metabolism-involved gene, glyceraldehyde-3-phosphate dehydrogenase (*GDPDH*, s355.g10357; average FPKM value: 2499), an important enzyme of energy and carbon molecule supply (catalyzes the conversion of glyceraldehyde 3-phosphate to glycerate 1,3-bisphosphate) was included. The 3’ untranslated regions (UTRs) are involved in mRNA stabilization and, hence, terminators were assumed to play essential roles in determining the stability and frequency of exogenous DNA expression. Thus, the 3’ flanking region of the alpha tubulin gene (*aTUB*, s182.g5608; average FPKM value: 1247) was chosen as the terminator (Fig. 3B and Table S1). The 3’ flanking sequences of the chloroplast genome encoding *PSBA* gene (s001.27891-29028) was used as a control.

To improve the translation efficiency, two factors were considered. *First*, a translation initiation site was arranged, via primer design, as the preferred Kozak consensus sequence in IMET1 (ACC*AUG*; where *AUG* refers to the starting codon) (Table S2). *Second*, the hallmark of the IMET1 nuclear genome was a codon bias favoring C or G at the third position (Table S3). Therefore, the selection marker gene *BLE* was completely re-synthesized in accordance with the nuclear codon usage of IMET1, and designated as *eBLE*.

The relationship between the expression circuit structure and the transformation efficiency was defined by assembling these building blocks into ten vectors (Fig. 3C). We also constructed vectors driven by an IMET1 endogenous *HSP70A* (g5260) promoter where the homologs were routinely used to drive constitutive gene expression in the nucleus of *Chlamydomonas* and several other microalgae [19, 35, 36].

### Characterization of assembled transgene circuits

**Fig. 4:**
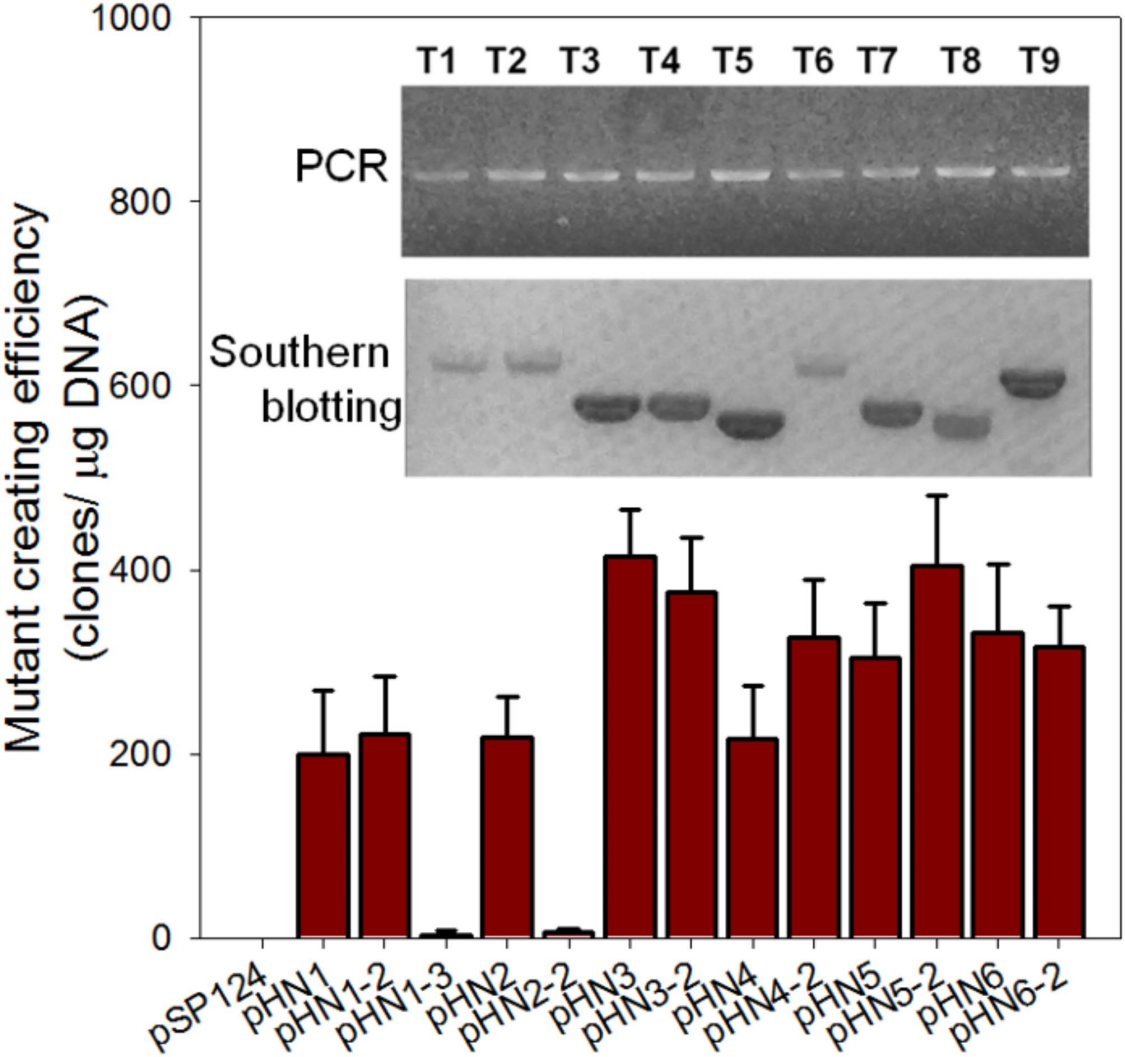
Transformation efficiency comparison of different transforming cassettes. The product generated with primer pair eBLE-F/R in the PCR analysis of transformants. Southern blots were probed with an DIG-labeled DNA covering the complete eBLE coding sequence of 387 nucleotides for the southern blotting analysis

Thirteen assembled vectors were one-by-one delivered into IMET1 cells. Zeocin (2.5 µg/L) was used to select transformants, which were further confirmed via genomic PCR and southern blotting (Fig. 4 inset). The transformation efficiency in IMET1 of exogenous promoters differed significantly from that of endogenous promoters. Vector pSP124 (harboring a *BLE* gene driven by an endogenous *Chlamydomonas RBCS2* promoter) achieved a high frequency of *Chlamydomonas* transformant recovery [40]. In contrast, no IMET1 transformants could be recovered for pSP124.

High transformation efficiencies (ranging from 217 ± 57 to 414 ± 102 transformants/µg) were achieved for the vectors assembled by promoters from the trimmed TOP 100 library (i.e., *VCP* g2383, *ATPASE* g335, *LHC* g240, and *GDPDH* g10357). The transformation efficiency of pHN3 was 138-fold higher than that of pHN1-3, which was designed empirically. However, the vectors harboring each of the four promoters exhibit only slightly variation (Fig. 4). The adoption of alternative terminators had negligible effect on the transformation efficiency (Fig. 4). Similar efficiencies of 200 ± 70 and 218 ± 44 transformants/µg DNA were realized for pHN1 (harboring *BLE*) and pHN2 (harboring *eBLE* with the remaining skeleton identical to pHN1), respectively (*p* value = 0.53; Fig. 4), probably due to a lack of rare codons in *BLE* (Figure S2). The results suggest that the merits of using codon-optimized expression cassettes will be accentuated when rare codons occur in the to-be-expressed proteins. Moreover, in this case, the difference of codon usage between *eBLE* and *BLE* should have no impact on the driving-efficiency evaluation among circuit assemblies.

### Application of validated vectors in high-throughput creation of the insertion mutant library

**Fig. 5:**
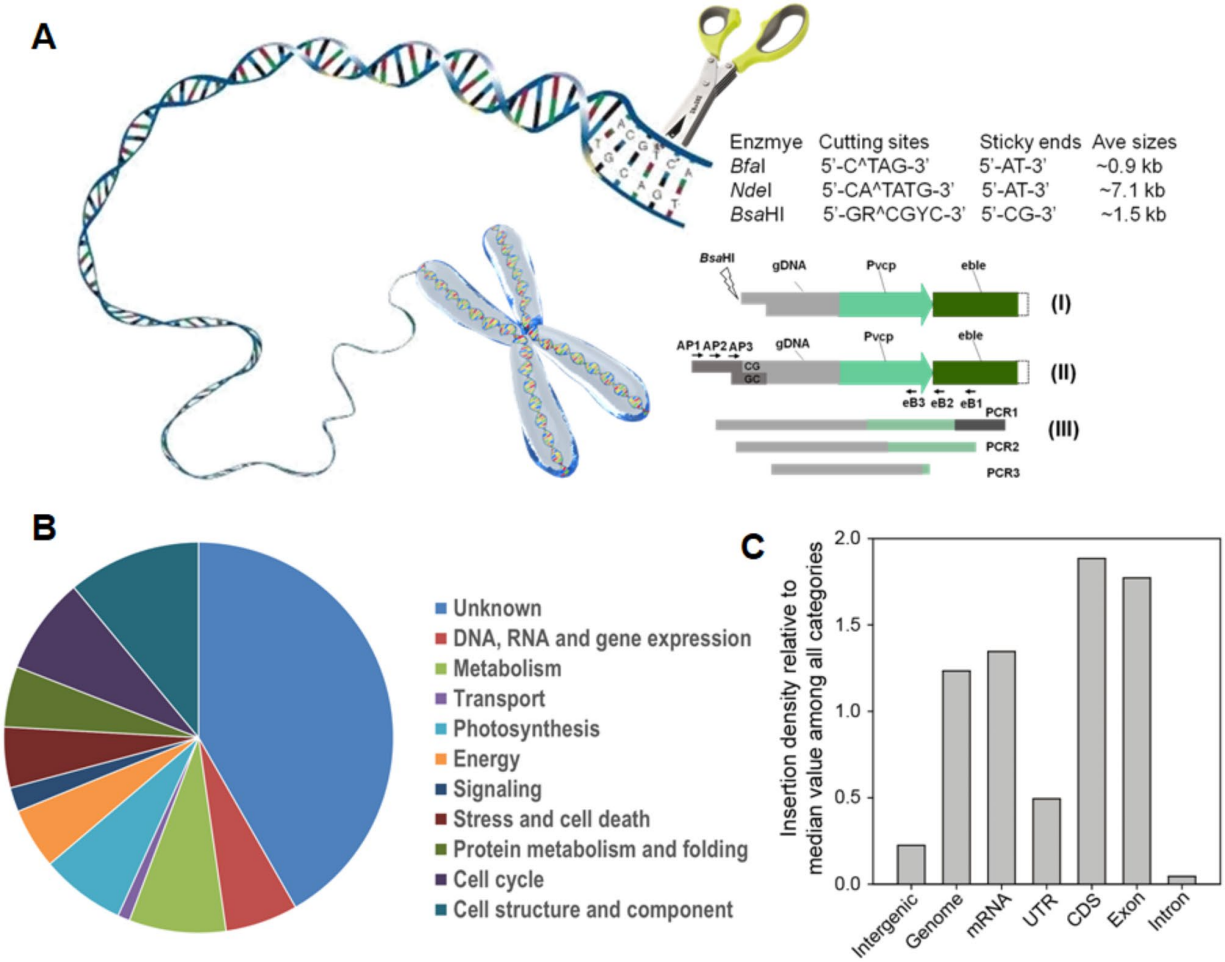
Distribution of *eBLE* insertion sites at a genome scale of the mutagenesis library. (**A**) Schematic of an adapter ligation-mediated PCR method for amplifying the flanking sequence tag of insertions. The steps show the (I) digestion of genomic DNA with *Bsa*HI, (II) preparation of the GC-adapter and generation of target DNAs composed of a known adaptor sequence and an unknown genomic flanking sequence, and (III) nested PCR with adaptor-specific forward primers (AP1, AP2, and AP3) and *eBLE*-DNA-specific reverse primers (eB1, eB2, and eB3). Enzymes *Bfa*I and *Nde*I produced a 5’-AT-3’ overhang with average restriction fragment sizes of 0.9 kb and 7.1 kb, respectively, while *Bsa*HI produced a 5’-CG-3’ overhang with a size of 1.5 kb. (**B**) Distribution of **B** inserted genes in functional categories. (**C**) Distribution of insertion sites based on gene features (5’UTR, CDS, intron, 3’UTR, and intergenic regions)

Employing pHN3, an insertion mutant library including approximately 10,000 clones was created. To assess the library, a customized ligation-mediated PCR (LM-PCR) was devised for IMET1-mutant amplification of the flanking sequence tags (FSTs) on *eBLE*-DNA insertions (Fig. 5A). The cutting frequencies of different restriction enzymes or their assemblies in the IMET1 genome were determined through an in silico analysis. This analysis reveals that, compared with *Nde*I (5’-CA^TATG-3’; average restriction fragment size: ~7.1 kb) and *Bsa*HI (5’-GR^CGYC-3’; average restriction fragment size: ~1.5 kb, see Fig. 5A), *Bfa*I (5’-C^TAG-3’) digested more frequently in IMET1 genome (average restriction fragment size: ~0.9 kb). A combination of *Bfa*I and *Nde*I digestion resulted in an average fragment size of 0.8 kb. Therefore, *Bfa*I, *Bfa*I/*Nde*I and *Bsa*HI were used for subsequent investigations. The ligation efficiency of enzyme-digested cohesive ends and designed adaptors was then probed. Enzymes *Bfa*I and *Nde*I produced a 5’-AT-3’ overhang while *Bsa*HI produced a 5’-CG-3’ overhang. Four pairs of adaptors (with a 5’-AT-3’ overhang or with a 5’-GC-3’ overhang) were designed (Table S4). Compared with that based on the 5’-GC-3’ overhang (*BsaHI* digestion), protocols based on the 5’-AT-3’ overhang (i.e., *Bfa*I or *Bfa*I/*Nde*I digestion) were less successful in flanking region retrieval. Therefore, the 5’-GC-3’ overhang was employed to produce 75.0% retrieval efficiency in the second-round PCR (PCR2; see Fig. 5A). The retrieval efficiency was optimized (up to 80.0%, see Table S4) by designing an additional pair of nested primers, which yielded increased band sharpness. For each selected mutant, the *eBLE*-gDNA cassette amplicons were trimmed and generated final FSTs (comprising a genomic sequence only), with 85% exhibiting high levels of homology to genomic sequences. Altogether, a robust flanking sequence retrieval method is established for IMET1 characterization of the insertion sites.

The mutagenic distribution and diversity of the pHN3 mutant pool was assessed via LM-PCR, producing 6,366 *eBLE*-DNA/genome junction sequences (Supplementary Dataset 5). The integration site of each *eBLE*-DNA was located by aligning each junction sequence with the IMET1 genome. Apparent PCR plate cross-contaminants were excluded. A conservative set of 5,707 high-quality *eBLE*-DNA integration-site sequences, representing 5,707 disruptions, were mapped onto the genome sequence. In addition, we determined the applicability of the sequence-indexed *Nannochloropsis* insertion mutant collection to functional analysis, by characterizing the FST of clones, which are randomly selected from the mutant pool. The insertion sites of all characterized single mutants in the mutant pool were identified via deep sequencing (Supplementary Dataset 6). A bias for insertions of a functional category of unknown genes in the mutant library (41.5%; Fig. 5B) was revealed. The preference was examined for FSTs within particular genetic elements, including UTRs, coding regions, exons, introns, mRNA, and intergenic regions (reads mapped to plastid genes were excluded). A highly non-uniform chromosomal distribution of integration events was observed (Fig. 5C). Preferred sites of FST or hot spots and cold spots occurred in the coding sequence (CDS) regions and in the introns, respectively. When genes were normalized by median value among all categories, we detected a bias toward insertions in gene regions. As for mRNA, we observed a significant bias against integration events in favor of CDSs versus UTRs and exons versus introns (Fig. 5C).

## Discussion

Although synthetic biology has been successfully applied in various bacteria, yeasts, and higher plants, by characterizing and collecting reusable and standard biological parts [41–45], microalgal synthetic biology remains in a stage of infancy. Attempts have been undertaken in only a few transformable laboratory-model microalgal species [46–48]. Many important microalga species with industry-scale production potentials remain genetically intractable. Moreover, even for transformed microalgae, hurdles associated with gene delivery efficiency and integration or stability have rendered the available transformation system impracticable, mainly due to the fact that many of the tools used for metabolic manipulation are developed based on experience-dependent strategies. Therefore, most of these tools are (rather than being universally applicable) pertinent to specific cases (e.g., certain species or strains) only. This lack of tool and technique transferability between microalga species stems primarily from an incomplete understanding of regulatory elements. However, the high-throughput sequencing of microalgal genomes has progressed at an ever-increasing pace. The increasing availability of genome information allows identification of species-specific biologically functional parts, modules, and systems and helps in the development of customized or universal tools for microalga engineering.

In this study, a full time-course dynamics of transcripts and their isoforms were revealed via deep sequencing. Quantitative genome-wide comparison of genes facilitates the delineation of regulatory mechanisms as well as an in silico prediction and accurate identification of promoter regions. TOP 100 genes (average FPKM value: ~3-fold and ~ 18-fold higher than that of TOP 1000 genes and all genes, respectively; see Figure S1) were identified and collected. Moreover, the use of trancriptomic analysis has yielded rapid methods of sequence-based structural annotation, which involves the identification of featured modules and is crucial for the correct expression of delivered genes. A series of expression circuits was devised and in vivo assessments of the corresponding relative activities revealed high transformation efficiencies. Additionally, the compatibility of promoters and terminators (i.e., the promoter-terminator pattern) is rarely explored in microalgal engineering. Investigating this aspect through in silico and in vivo approaches may offer a strategy to enhance gene expression in *N. oceanica*.

This knowledge-driving strategy provides several potential advantages over the traditional method for microalgal transformation system establishment. First, in silico prediction and investigation of regulatory elements increase the likelihood of discovering active regulatory parts, which are crucial for a successful transformation system. The transcription of foreign genes and/or transgenes are typically expressed in a 5’ promoter and a 3’ terminator cassette. Conventionally, marker and reporter genes under the control of strong constitutive promoters from evolution-similar species have been applied for the transformation of microalgae [12, 49]. Adherence to this empirical rule should, however, be approached with caution, as inconsistencies may arise. For example, heterologous *CaMV35S* and *SV40* promoters drive transient expression in some algal species, but inadequate recognition of the heterologous promoter region and lack of adequate regulation are typical [50]. In this work, transformation of *Nannochloropsis* via the *Chlamydomonas* vector pSP124 was unsuccessful. Thus, the transformation efficiency of a microalgal expression system based on the conventional strategy is occasionally unstable and impractical for gene expression and is, hence, inadequate for the creation of a mutagenesis library. *Second*, a genome-scale preview of gene structure sheds light on species-specific regulatory mechanisms at several layers (e.g., transcriptional and translational levels). The computational analysis of biological parts renders genetic engineering more rational (than engineering without aid of this analysis) and helps to find, characterize, and standardize promoters, UTRs, terminators, and codon preferences. Empirically, heat shock protein and tubulin were considered highly expressed proteins in organisms (e.g., *Chlamydomonas* sp.). Their promoters were routinely used to drive constitutive gene expression in the nucleus of *Chlamydomonas* sp. and several other microalgae [19, 35, 36]. However, several homologs with unknown expression levels are present in the microalgae of interest and therefore the selection of driving promoters for vector design is difficult. Hence, prior to the application this genome-based strategy, we also constructed vectors driven by IMET1 endogenous promoters of gene encoding *HSP70A* (g5260), *HSP70A* (g302), tubulin beta chain (*β-TUB*, s026.g1388), and tubulin delta chain (*δ-TUB*, s127.g4421). Application of promoters in IMET1 for various metabolic engineering experiments has nevertheless only been successful for *HSP70A* (g5260) and *β-TUB* (g1388) [51–53], and unsuccessful otherwise. The driving efficiency at the protein level has yet to be assessed and the driving activity of promoters may vary across experimental conditions. However, a practicable transformation is more likely for promoters with in silico-predicted high activities than those with low activities. *Third*, a cross-species genome comparison elucidates the universal rules of the regulatory mechanisms in microalgae. Nearly 30 complete genomes are currently available to the public for eukaryotic microalgae of various species, and many more will be sequenced in the next few years. The investigation of these data enables identification and characterization of reusable and novel biological parts. Profiling and identification of BioBrick parts using RNA-Seq technique helps the construction of a scalable transgenic toolbox. Furthermore, the assembly and engineering of individual parts with defined functions from libraries of standard interchangeable parts permit design and development of generalizable engineering tools.

Gene silencing has recently been used to determine the role of predicted genes comprising the *Nannochloropsis* genome [51]. The RNA interference (RNAi) method suffers, however, from several drawbacks including the lack of stable heritability of a phenotype and variable levels of residual gene activity [54]. Targeted gene replacement via homologous recombination or CRISPR/Cas9 is extremely facile in some eukaryotic microalgae [55, 56]. However, owing to the relatively low efficiency of this technique in specific strains of *Nannochloropsis* spp. and other documented microalgal species, high-throughput creation of a genome-wide gene disruption set is prevented [52, 57–59]. The ability to create loss-of-function mutations for each gene is essential for the functional analysis of these completely sequenced genomes. Hence, we determined the suitability of the assembled transgene circuits by creating and characterizing a gene-indexed loss-of-function mutation library. Altogether, the genetic toolbox provided here plus the set of genomes, transcriptomic investigations relevant to oleaginousness, and advantages of *Nannochloropsis* will facilitate the (*i*) development of versatile engineering tools for genome engineering [51, 52, 60] and (*ii*) studies of various cellular mechanisms, including photosynthesis [21, 26], carbon partitioning [27], lipid metabolism [23], sterol metabolism [30], and phytohormone function [28, 29].

## Materials and methods

### Algal culturing, sampling, and RNA-sequencing

*Nannochloropsis oceanica* IMET1 was inoculated into a modified F/2 liquid medium under continuous light (∼50 µmol photons m^− 2^ s^− 1^) at 25 °C [31]. The cells were grown in liquid cultures under continuous light (∼50 µmol photons m^− 2^ s^− 1^) at 25 °C and aerated by bubbling with a mixture of 1.5% CO_2_ in air. Mid-logarithmic phase algal cells were collected and re-inoculated in a fresh medium. Cultures started with a same-cell concentration (OD_750_ = 2.0) and collected for RNA isolation after 3, 4, 6, 12, 24, and 48 h. Three biological replicates were established for mRNA-Seq analysis. RNA extraction, cDNA synthesis, transcriptome library preparation, and sequencing were undertaken as previously described [23].

### Regulatory element detection and in Silico analysis

Gene expression was measured as the numbers of aligned reads to annotated genes using the Cufflinks (version 2.0.4) software and normalized to FPKM values. Using MultiExperiment Viewer 4.8 with Euclidean distance, the TOP 1000 differently expressed genes were grouped, via k-means clustering, into 10 clusters based on their temporal expression patterns. Prior to clustering, gene expression data were preprocessed via log_2_ transformation and z-score normalization, to ensure comparability across samples. The optimal number of clusters (k) was determined using the Elbow method and Silhouette analysis, evaluating within-cluster sum of squares (WCSS) and cluster cohesion. Clustering was performed using the k-means algorithm with Euclidean distance as the similarity metric. The algorithm iteratively assigned genes to clusters by minimizing intra-cluster variance until convergence. To enhance robustness, clustering was repeated 100 times with different initial centroid seeds, and the most stable clustering solution was selected. Following grouping was conducted by KEGG [61]. The results were cross-validated with Gene Ontology (GO) enrichment and protein-protein interaction (PPI) networks from STRING to ensure consistency in functional classification. The featured modules in the promoters (e.g., TATA box, CAAT box, GC box, and OCT element) were analyzed at PlantCARE (http://bioinformatics.psb.ugent.be/webtools/plantcare/html/). Furthermore, Kozak consensus sequence detection was performed with a custom-developed R language.

### Biological part design, cloning, and assembling

The IMET1 endogenous promoter *HSP70A* (s162.g5260) and terminator *PSBA* (s001.27891-29028) were amplified using genomic DNA with specific primers (HSP70A-F1, HSP70A-R1, PSBA-F1, and PSBA-R1; Table S2) and sub-cloned into a pBluescript SK vector (Stratagene) using *Kpn*I, *Xho*I, and *Bam*HI, *Sac*I sites, respectively. The *BLE* gene was amplified from vector pSP124 using primers BLE-F and BLE-R and ligated into the above vector between the *Xho*I and *Eco*V sites. Subsequently, the obtained vector was nominated as pHN0. Using vector pHN0 as the template, the Phsp70A-BLE-TpsbA cassette was cloned with specific primers (HSP70A-F2 and PSBA-R2) and digested with *Kpn*I and *Sac*I. The digested Phsp70A-BLE-TpsbA cassette was sub-cloned into vector pHN0 digested by *Kpn*I and *Sac*I. The resulting vector was nominated as pHN1. Vector pHN2 was constructed by substituting the coding sequence of the *BLE* gene in pHN1 with codon-optimized *eBLE* (synthesized by Invitrogen). The promoters of gene *VCP* (g2383), *ATPASE* (g335), *LHC* (g240), *GDPDH* (g10357) were amplified and assembled into pHN2 digested by *Acc*I and *Xho*I, respectively. The preferred Kozak consensus sequence ACC was inserted before the start codon ATG, and the resulting vectors were nominated as pHN3, pHN4, pHN5, and pHN6, respectively. Afterward, the *α*-*TUBULIN* (g5608) terminator was amplified and assembled into pHN3, pHN4, and pHN6 digested with *Bam*HI and *Hind*III, yielding pHN3-2, pHN4-2, and pHN6-2. The PSBA terminator of pHN5 was digested with *EcoR*I and *Sac*I and substituted by the *α*-*TUBULIN* (g5608) terminator, resulting in pHN5-2 (the corresponding primers are listed in Table S2). The cloning steps were all performed in *Escherichia coli*, in accordance with Green and Sambrook [62].

### Transformation, mutant library generation, PCR screening, and Southern blotting

Cells at log phase were collected and transformed by means of a previously described method [33]. Four replicates were set for each vector. After the electroporation process, the cells were plated on the F/2 agar plate containing 2.5 µg/L zeocin and incubated at 25 °C under continuous illumination at ~ 50 µmol photons m^− 2^s^− 1^. Transformed colonies became visible after approximately three weeks. Individual colonies were removed and transferred to fresh F/2 media. The occurrence of *BLE* or *eBLE* genes in the resulting independent transformant lines was evaluated. Genomic DNA was isolated and analyzed via PCR using the (*e*) *BLE* primers listed in Table S2.

For southern blotting, genomic DNA was isolated from exponential phase cells ground in liquid nitrogen with a chilled, sterile mortar and pestle. Extraction was performed using a Plant Genomic DNA Mini Kit (Omega). Five micrograms of total DNA was digested completely with *Kpn*I, and the resulting fragments were separated on 0.8% (w/v) Tris-acetate EDTA agarose gels and then transferred to nylon membranes via capillary action. The probe (0.3 kb) was amplified via PCR using pHN2 vector as a template. Afterward, the PCR product was column purified, and a labeled probe was generated through incubation with digoxigenin-dUTP. The DNA transfer, fixation, hybridization, and immunological detection procedure was performed in accordance with the instruction manual (Roche).

### Flanking fragment retrieval

An adaptor-ligation PCR method was customized for IMET1 using a modified version of a previous approach [63]. This method consisted of the following steps (1) extraction of genomic DNA from transgenic algae and restricted digestion using the *Bsa*HI enzyme, (2) ligation of the *Bsa*HI adapter to the genomic DNA, (3) first-round PCR with primers AP1 and eB1 specific to the *Bfa*I adapter and *eBLE*-DNA, (4) second-round PCR with primers AP2 and eB2, (5) sequencing of the PCR2 products, or if necessary, third-round PCR using primers AP3 and eB3, and (6) identification of *eBLE*-DNA flanking region(s). With the exception of eB3, which is promoter-dependent, the same set of primers is applied to each of the transforming cassettes.

### Highly parallel DNA sequencing and flanking sequence analysis

The *eBLE*-gDNA cassette PCR amplicons were analyzed via pyrosequencing on a 454 Life Sciences Genome Sequencer FLX Titanium (GS-Titanium; 454 Life Sciences, Branford, CT, USA) that produced (on average) 250 bp long reads. To assess the accuracy of the *eBLE*-gDNA cassette PCR amplicon-based measurement of insertion site diversity and distribution structure, we created two mutant genomic DNA pools. Library-A and Library-B consisted of insertion-site-known individuals and a collection of insertion-site-unknown subjects, respectively. The insertion sites of the individuals from both libraries were characterized via tail-PCR. Two rounds of nested PCR were performed, and shotgun pair-end libraries of total PCR amplicon were then prepared. Each metagenomic DNA library was subsequently sequenced and an unspecified amplification in Library-A was set as background noise. These noises were subtracted from the mutant pool.

The flanking sequences of the 5’ terminus of the transforming cassette were retrieved. The raw data of the *eBLE*-gDNA cassette PCR amplicon was composed of a cassette sequence and the genomic flanking sequence. Afterward, the cassette sequence was removed from the amplicons. The trimmed reads (average length: 367 bp) were independently aligned to the IMET1 genome, allowing one mismatch to the cassette sequence; non-unique alignments were discarded. Reads aligning to the same position were combined as single insertion flanking sequences. Flanking sequences without a hit in the genome were discarded.

## Electronic supplementary material

Below is the link to the electronic supplementary material.

**Supplementary Figure 1:** The transcript abundance of genes in terms of the average FPKM value (Fragments Per Kilobase of exon model per Million mapped fragments). (**A**) Transcriptional pattern of the top 1000 transcripts grouped into 10 clusters. The transcriptional abundance of each gene was sorted from highest to lowest according to the FPKM values at six time points. Genes with FPKM values among the top 1000 at any of these points were designated as TOP 1000. (**B**) Average FPKM values of genes where the regulatory regions are used for transforming the cassette construction. (**C**) Distribution of genes in functional categories within each cluster. C1 to C10 denote the 10 clusters of the TOP 1000 genes. Top 100 denotes genes among the TOP 1000 with FPKM values > 319 at all six time points. Abbreviation: VCP: violaxanthin/chlorophyll, a binding protein, ATPase: v-type ATPase, LHP: Light harvesting protein, GDPDH: glyceraldehyde-3-phosphate dehydrogenase, a-TUB: α-tubulin, HSP70A: heat shock protein 70 A

**Supplementary Figure 2:** Comparison of codon usage by *BLE* and codon-optimized *BLE* (*eBLE*). Arrows indicate codon-optimized amino acids. The numbers before and after each dash represent the codon usage ratio encoding the corresponding amino acid in BLE and eBLE, respectively

**Supplementary Dataset 1.** Clusters of the TOP 1000 genes

**Supplementary Dataset 2.** Expression pattern of the TOP 100 genes

**Supplementary Dataset 3.** Occurrence and distribution of featured modules in promoter regions of the TOP 100 genes

**Supplementary Dataset 4.**The nucleotide acid around the initiation codon from the mRNAs associated with the trimmed TOP 100 genes.

**Supplementary Dataset 5.**Flanking sequence data of the insertional *N. oceanica* IMET1 mutant pool.

**Supplementary Dataset 6.** Random distributions of *eBLE*-DNAs determined via the individual clone-based validation method and mutant-pool validation method.

**Supplementary Tables: Table S1.** Transcriptional dynamics of genes where the promoter or terminator regions were used for vector construction.**Table S2. **Primers used for expression vector construction.**Table S3.** Codon usage of *N. oceanica* IMET1. **Table S4**. Sequences of the oligonucleotides used for adapter ligation-mediated PCR method customized for mapping of *eBLE*-DNA inserts in the *N. oceanica *IMET1 genome.

## Data Availability

No datasets were generated or analysed during the current study.
