# Supplementary figures and images for: Systematic approach for dissecting promoters and designing transform systems in microalgae
Source: Microb Cell Fact. 2025 May 29;24:127. doi: 10.1186/s12934-025-02700-5 (PMC12121064; doi:10.1186/s12934-025-02700-5)

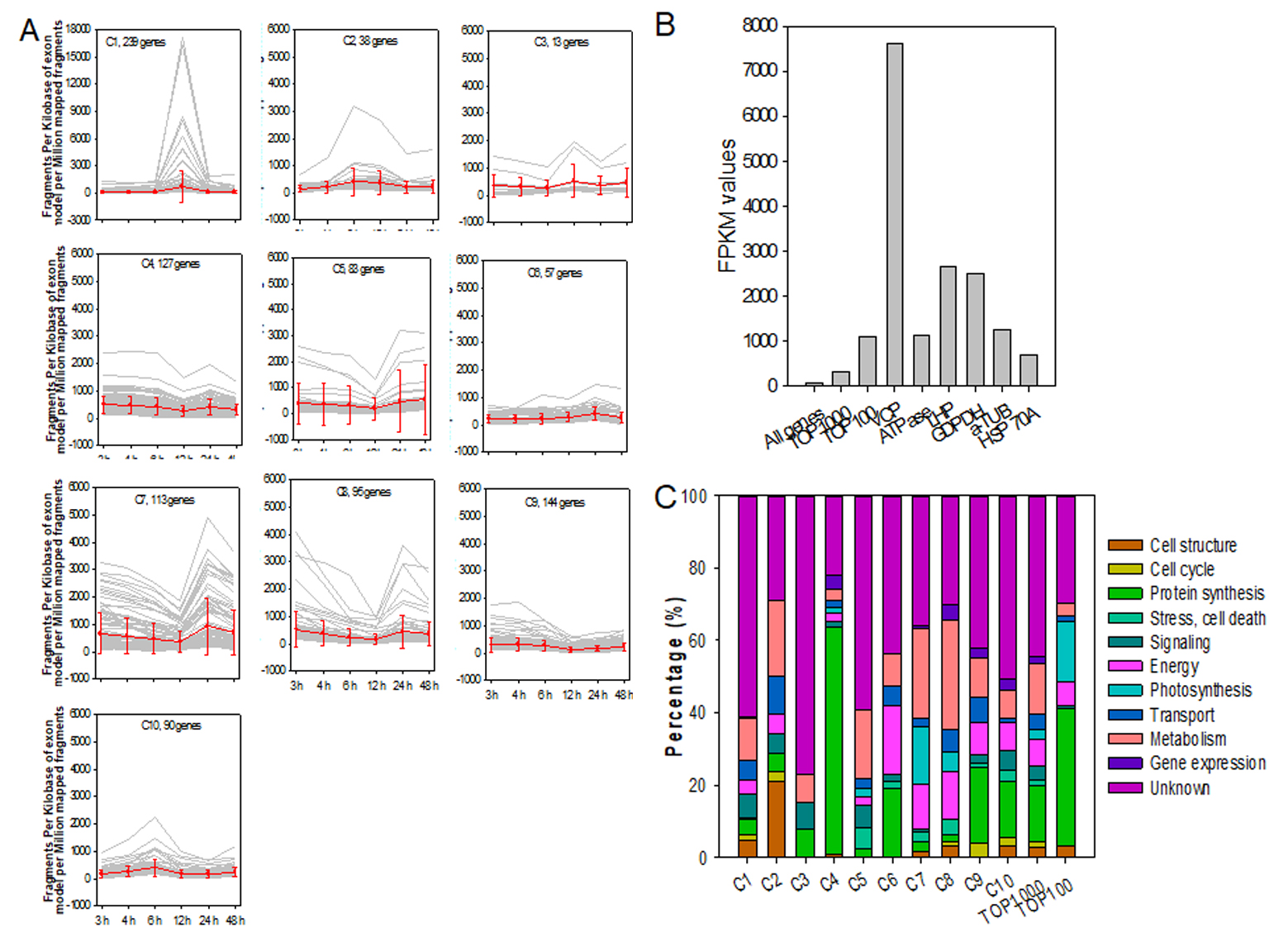

Supplement: Supplementary file 1 — Supplementary Figure 1: The transcript abundance of genes in terms of the average FPKM value (Fragments Per Kilobase of exon model per Million mapped fragments). (A) Transcriptional pattern of the top 1000 transcripts grouped into 10 clusters. The transcriptional abundance of each gene was sorted from highest to lowest according to the FPKM values at six time points. Genes with FPKM values among the top 1000 at any of these points were designated as TOP 1000. (B) Average FPKM values of genes where the regulatory regions are used for transforming the cassette construction. (C) Distribution of genes in functional categories within each cluster. C1 to C10 denote the 10 clusters of the TOP 1000 genes. Top 100 denotes genes among the TOP 1000 with FPKM values > 319 at all six time points. Abbreviation: VCP: violaxanthin/chlorophyll, a binding protein, ATPase: v-type ATPase, LHP: Light harvesting protein, GDPDH: glyceraldehyde-3-phosphate dehydrogenase, a-TUB: α-tubulin, HSP70A: heat shock protein 70 A [file 12934_2025_2700_MOESM1_ESM.jpg]

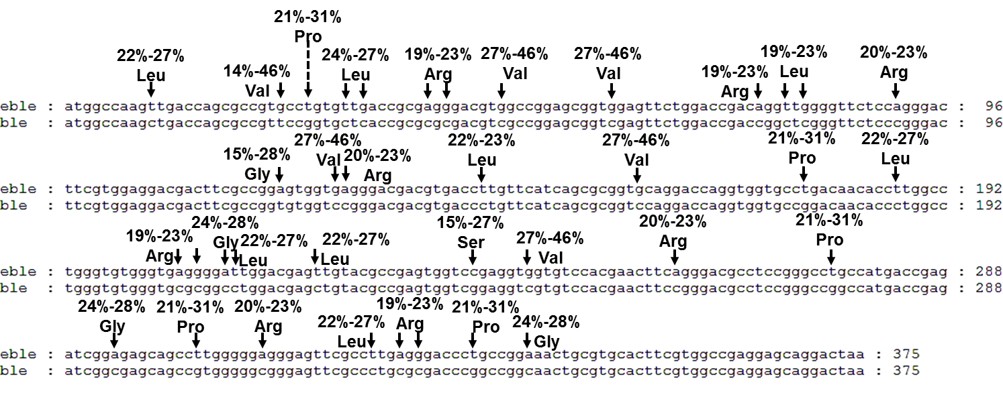

Supplement: Supplementary file 2 — Supplementary Figure 2: Comparison of codon usage by BLE and codon-optimized BLE (eBLE). Arrows indicate codon-optimized amino acids. The numbers before and after each dash represent the codon usage ratio encoding the corresponding amino acid in BLE and eBLE, respectively [file 12934_2025_2700_MOESM2_ESM.jpg]
