# Supplementary material for: Systematic approach for dissecting promoters and designing transform systems in microalgae
Source: Microb Cell Fact. 2025 May 29;24:127. doi: 10.1186/s12934-025-02700-5 (PMC12121064; doi:10.1186/s12934-025-02700-5)
Supplement: Supplementary file 9 — Supplementary Tables: Table S1. Transcriptional dynamics of genes where the promoter or terminator regions were used for vector construction.Table S2. Primers used for expression vector construction.Table S3. Codon usage of N. oceanica IMET1. Table S4. Sequences of the oligonucleotides used for adapter ligation-mediated PCR method customized for mapping of eBLE-DNA inserts in the N. oceanica IMET1 genome. [file 12934_2025_2700_MOESM9_ESM.docx]

**Molecular investigation of** Δ**g5581 mutant**

**FST (AP2 and eB2 PCR product)**

*ACAAGAATTGGGTCAGTACTCCACCGCTCCGGCCACGTCCCTCGCGGTCAACACAGGCACGGCGCTGGTCAACTTGGCCATCTCGAGGGTCTTTGGTTGTGATATTGTTTTGTTACTGTGGTATGGGTGAAGGTTGGGAGCGGTGAGTGAGTCGTCGAAAGTTCTTTGTGTTTTCTGTGGCTTTCGCTGTCCCTTTTCCATGTCGCAGAAGAACGAAAATGACACAAGGCTACAGCGGGGGCACGATGCATCGTCACCGTAACTGAACACGTAGTGTTATCAAGACGCATCGGCAGGGCGGGCACGCGCCATGTGGGTGAAGGCATCTTTAAAGACGAAGCCGCTCTCCGCTCCACAGCCACACACACAGCACGTTCTCTGCCTGAAGACGAAAATTTGACGCTTCGCAGAGGCTTGGAGTCGTGCCAGCAGCCCCTGCTTTGTATTCTACATTGAGTGGGACAGGGTTTACGAGA*gtagacatcgatggtacccagcttttgttccctttagtgagggttaattgcgcgcttggCGTAATCATGGTCATAGCTGTTTCCTGTGTGAAATTGTTATCCGCTC*CCCAAGGGGTTTCC*AGCTCCAGGGCTTGAAAAAGAAGCAGTCCGCACTGTCCGACCTCCCGGCTCGGGCAAAATTATTGTATAAAACATCCGGACAACTTGCTTGTACATTTAAAAAAATTTCCAATATGATTCATGACGATGATCTTATGGAGATCCCTTTACGTGCTTTGTACGTAAAAAACTGAGATCACTTTGAGGTGGCATCAGCACACCTAATGTATACAACATGTTTTGACCTCTAATTTTTTCCAGACTCTCATAGAGGCTGCCCGGAGGAGCTACGATACCTCATCTCGCGCCAGAGATCTTCTGACCTTGATGTGCTGAGTGCACTCCTGGATGAATCACATCTCAATAGGATGCACGTCTGAGCCGTTCATCGTCCGAGTAGACACTATGCTCGTGGACTCCCCCCGGCCTCTCGGATTCATACTCCAGCTCCGCCCCTCAGCCGCACAAGCGTGACCGATATTCCGGTCAGGTGCTCCAAGAGACGATTCCCCTCTCCCCCAAGAGTGTGCCTCCGGATTCAGGTTTAGGCAAGTTCTGGAGCGGGTTACGCATCTCACAGGGCCCCTCACACACACAAAA

***eBLE*-DNA sequence of mutant Δg5581**

ATTTTTGAAGATTGAGAAGATGCTTAAGGAGGAGGAAGTGAGGGAGATCGCTTTTCGTGCTAAGTACGGCCCAGCCTGGGATAAGTTTGGGGAGCCATCCGCACAGCTCATGGCTACTACATGGAAGGAGGTCTCACATTTCCAGACTCTCTTTGCGGCTGCCCAGAGGAGCGACGATTACCTCATCTCGCGCCTGCGCTCTCCTGAACTTGATGGCCTGAGTGCACTGCTGGATGAATCGCGTCGGAATTTGGATGCACGTTTGGCacgttgtaaaacgacggccagtgagcgcggtaatacgactcactatagggcgaattggagctcccgcggaagcttcgcaacgtccttatgaagaaccaggtttctatctagaaagacaagaaactgaaggtcgtaacatccgttacactattcatagttatgttgttaacaaatatcctcctggagaacgttacgttttataattcttaaataagtaacaactagttcatcaatttgatgaactagttgttacttacttactttattagatcaatttttataatttactagaatgtatagataccgcgttcactttatttttgagtaaaattaaattccatttgaaatcgttcgcttatcttttttacctatccatgaagttagtttgagtataatacttagacgctcagttaagttggatagagcaacaatactataaaatagtatttttacacttaatttagcaagtatataaaaaagtctaaccgtttcatttttacttaattaaattagatcaaaaaaattattacacatgagtgattaaaaatcactcatgtgtaattaaatattcttaagcattaacagcaggagcgtttaatgcaactggaagaacattagatacggggatcccccgggctgcaggaattcgatatcttagtcctgctcctcggccacgaagtgcacgcagtttccggcagggtccctcaaggcgaactccctcccccaaggctgctctccgatctcggtcatggcaggcccggaggcgtccctgaagttcgtggacaccagatcggacactcggcgtacaactcgtccgatcccctcacccacacccaggccaaggtgttgtcaggcaccacctggtcctgcaccgcgctgatgaacaaggtcacgtcgtccctcaccactccggcgaagtcgtctcagagtcctggagaaccccaacctgtc*ggtccagaactccaccgctccggccacgtccctcgcggtcaacacaggcacggcgctggtcaacttggccatctcgagggtctttggttgtgatattgttttgttactgtggtatgggtgaaggttgggagcggtgagtgagtcgtcgaaagttctttgtgttttctgtggctttcgctgtcccttttccatgtcgcagaagaacgaaaatgacacaaggctacagcgggggcacgatgcatcgtcaccgtaactgaacacgtagtgttatcaagacgcatcggcagggcgggcacgcgccatgtgggtgaaggcatctttaaagacgaagccgctctccgctccacagccacacacacagcacgttctctgcctgaagacgaaaatttgacgcttcgcagaggcttggagtcgtgccagcagcccctgctttgtattctacattgagtgggacagggtttacgaga*gtagacatcgatggtacccagcttttgttccctttagtgagggttaattgcgcgcttggcgtaatcatggtcatagctgtttcctgtgtgaaattgttatccgctc*CCCAAGGGGTTTCC*CCAGCCGTCGTCGGAATCGTCGCCCGTGCTGGTGGACTCATCAAATCTCTCGGCGGCCATGCTACGGCTGGCCCGGTTACTGGACGAGCGAGACGCCCTTCTGGTGCGGATTAAGGCGGAGCCTGATGCAATGTATGTGCGGTTAAAGGGACGGCTGTTAGCCGGCGAGCTGGTAAACGAGGCCGTGCCTCCGGCGATGGCCTCTGTGGGGGAGCTCAAGAGTGCATTGGCGGCGTCGATTGCAGGACAAGGGCCCTTAGTGGAGGAGATTTTGAGTGAGAACGAGCGATTTCAAGCAGG

>cDNA of g5581

ATGGATGGGCCATCCGAGGACTGTATTGGTGTGACACCTGTGAACAGTTCGTGTCTCGGGACGGCAATGGGGCACGTAATATCCTACGGGTGTACAGGAGTATGGTCCGTGGGGAAGCTCGTCCCCATGATCTGCGGTTTGGTCAACCGCGGCAGGATAAGCGGACACTCTATGTGTAGCGTTCCCGCTGACCTCCTCTTTTATCCTCCTTCCCCCCCTCCTCCTGGCCCTGTGTGGGCCGTCCGGGGACTTTGCCAGGCTCCCCCTCGTATGCAAGTCCTCGAAGACGTCAGGTTGCATGCGATAGTTCTAGAGAGATGTAGGAACTCAAAAGAACTAACCCCCACACACTCTCCGCCGCGAATCGACCCGGTTTCAAAAAGGGGCGAACTGAGTTCAAGGGTGCGTGACGGAAGAGGCTTTTCCGGAGTCCGCTTGTCTTATGCGCCACCGCGCAATCCACTGAAGCCCGACGTTAACGCACACATTAACCCATTCATGAATGAAAGTCAGGAGAGATGTGCCAAGCATTCTAGGAGGCGACAAGCGTCGTGTGCGAATCACGGCGGCTCAACATCCCGTCTTACCTGTGCCCTCCCCCACTGCCGCACAGTCACGACCGACCCATGCTACCCATTCCACTTAAGAGCAGCACGGGCGTTAATTTCAACCCTCTACGTGTCTATGTCTCAAACGACTCACCCTCATGCCCCATATACAGCCTCAGTTCTGAATACTGCGTCCGAAACTCCTGAGGTCCAGCGTGGATCTTCCACAGTCATCTATTTTCTTACGCACACCCATGTACAGGATGAAGCCGAGGCGGCGGAGGGTGACTTTAAGGCCCTCGATCGTATGCGGAATATGGCCATGCAAGCCCGCGCTGCCACGGACACCTCGGTCGCCACATTGCAAGCCTACTACGCCCAAGTGAACCGTCTTCACGGCATCTTTCCCATCAATACCGACCATATCCGCATCGCCTTTACCTGGCACGACGCCTTTCGCCCCACCAAGAAGTCTACAGAAATGGACTTGAACTTTGAGCGGGCAGGCGTACTTTTTAACATTGGGGCCGTCAAGAGCAACATTGCTGCATCGGCAGACCGGACGTCGGCCGAGGGAATGAAACTTGCTTGTCGGGAATTTCAGTTGGCGGCCGGCCTATTCAAGCACTTGCGCTGTCACGTAGTCTCGCAGCTGAATTGTAGCCTACCGTCGGAGCTCACCGAGGAGGGCTTGCTCATGGTGGAGAACATCATGTTGGCACAGGCCCAAGCCTGTTTTTACGAGAAGTCCGTGGTTGACCACCGGAGCGCCAGCTCAAGCATGAAAGCCTCTGTCGTGGCCCGGCTCGCAGCGCAGGCAGCTGAGATGTACAGCACTGCTCTCAAATACGGGGAGTCTCCAGCCATGGAGGCCGCAGGGCTTGACCGCTCCTGGCCTATTACGCTCCGTTTCCAGCAAGACTTTTACTCGGCCGCAGCGCAGTACTACCAGGCGGAGGCCTGCAAGGAGGCGGCCCAGGAAAATGGCAGTGGCTACGGAGAGGAGGTGACCCGTTTACGAATGGCTCAGACCTACATAGAAGTGGTGCTGGAGACCGCGGCAAGGCAACGCATGGGTCCTGCCATTATTGGGAAAGCCGAACTGTTGAAGGCCAAGATTGTGAAGAACAAAGCAGTGGCAGAGAAGGACAACAGTACCATCTATTTGGAAACGGTGCCAACCGAGGCCTCACTCAAGCCTGTCAATAAGACATGTATGGTTAAGGTCGTTGAGCCCGAGCCCTCTTTCCCATCCACTGGAACCTTGCAGCCCACCCTCTTTGCCAATATCCTTCCCAAGGCCATCAAGGAGGCGTTGACCGAGGCCGAGAAGCAGGTTGTTGCCCTAGCTGCAGCAATGGAGCAGGAGGGTCGGGAAGCATCCAAGGGGGCACGACAACAGCTGGCGAGAGCGGGTCTGCCTGGGTCCCTTCAAGCACACGAAAGCCCCGCCGGCCTCCCGGAGGCAACCTGGAAAAAGCTCCAGGGCCTGAGGGAGAAGCAGTCCACAGTGTCCGACCTCCAGGCTCAGGCAGAGGAATTGCAAAAAACATCTGGACAAGTTGCGGGGACATTTTTGAAGATTGAGAAGATGCTTAAGGAGGAGGAAGTGAGGGAGATCGCTTTTCGTGCTAAGTACGGCCCAGCCTGGGATAAGTTTGGGGAGCCATCCGCACAGCTCATGGCTACTACATGGAAGGAGGTCTCACATTTCCAGACTCTCTTTGCGGCTGCCCAGAGGAGCGACGATTACCTCATCTCGCGCCTGCGCTCTCCTGAACTTGATGGCCTGAGTGCACTGCTGGATGAATCGCGTCGGAATTTGGATGCACGTTTGGCXXXXXXCCAGCCGTCGTCGGAATCGTCGCCCGTGCTGGTGGACTCATCAAATCTCTCGGCGGCCATGCTACGGCTGGCCCGGTTACTGGACGAGCGAGACGCCCTTCTGGTGCGGATTAAGGCGGAGCCTGATGCAATGTATGTGCGGTTAAAGGGACGGCTGTTAGCCGGCGAGCTGGTAAACGAGGCCGTGCCTCCGGCGATGGCCTCTGTGGGGGAGCTCAAGAGTGCATTGGCGGCGTCGATTGCAGGACAAGGGCCCTTAGTGGAGGAGATTTTGAGTGAGAACGAGCGATTTCAAGCAGGGCGGAAGACCGACCCAGCGTTGTTAGAAAGAGATGCACTTGTGCAGCAGCTAGAGACGGGGGTGTTGCAGTGCCACGAGCTTCATGCCCAGTTGGCGGAAGGTCGGGAATTTTATGCTTCGGTAAGCAAGCGAATACAGCAGCTATTGCTGGTGACAGAAGATCAGCTTTACACCCAGGATATCCAACGTCGTGATTTTGAGGTGGAGCTCGGGCAGTCCGCCAATCGGAAGAAGCAAGAAAGTGATGATCAAGATGTGGCCAGGAAGCTTTTCGAGCAGCTCAACGTCCAGCAGCAGCAAGAAGGGGAAGGTGCCGCCACAACCGGAGACGCGCCTCATTTTGTGCCCCAGCCGCCATTTGGTGGTGGTGGACCGTCTCCGTCTGTCCCTCCTCCCCCACCTGCCATTAAGCCTCTTCCTCCTCCCCCCCATTCGCTCTCCACCCCAACTCCGGGGCCACCCCCTTCCTACGAGCAAGCTCAGGCTTTGCCGATCGCGCCTGCCTACACGGAGCAGGTCAAAATATCCCAGTTGGTTCAAATGGGTTTTCCAAAGGCACAAGCTCAGCATGCGCTGGTACAGGAGAGAGGGGATTTACAGGCGGCTCTCAATGTATTGCTCTCGGGCTGA

>gDNA of g5581

ATGGATGGGCCATCCGAGGACTGTATTGGTGTGACACCTGTGAACAGTTCGTGTCTCGGGACGGCAATGGGGCACGTAATATCCTACGGGTGTACAGGAGTATGGTCCGTGGGGAAGCTCGTCCCCATGATCTGCGGTTTGGTCAACCGCGGCAGGATAAGCGGACACTCTATGTGTAGCGTTCCCGCTGACCTCCTCTTTTATCCTCCTTCCCCCCCTCCTCCTGGCCCTGTGTGGGCCGTCCGGGGACTTTGCCAGGTCAAAAAACCTATCAAAGTCGCTAGAAAAACTCGGACGAGAAATAAGGGATGACAACAATTTATTGTTGGAAGGCTCCCCCTCGTATGCAAGTCCTCGAAGACGTCAGGTTGCATGCGATAGTTCTAGAGAGATGTAGGAACTCAAAAGAACTAACCCCCACACACTCTCCGCCGCGAATCGACCCGGGTGAAAATATCGCACCCGGCACCCACTCCAGGGCCGTGAGATATAATTTTGTGGACTTGTTTCATCAGTCCGGCGACTCGTTCAGTTTCAAAAAGGGGCGAACTGAGTTCAAGGGTGCGTGACGGAAGAGGCTTTTCCGGAGTCCGCTTGTCTTATGCGCCACCGCGCAATCCACTGAAGCCCGACGTTAACGCACACATTAACCCATTCATGAATGAAAGTCAGGAGAGATGTGCCAAGCATTCTAGGAGGCGACAAGCGTCGTGTGCGAATCACGGCGGCTCAACATCCCGTCTTACCTGTGCCCTCCCCCACTGCCGCACAGTCACGACCGACCCATGCTACCCATTCCACTTAAGAGCAGCACGGGCGTTAATTTCAACCCTCTACGTGTCTATGTCTCAAACGAGTATTCCAAGGTAAGGAGTATCGGAATTTAATGAGGGCTCCTCTAGTATACTTTCTACTCGTCTCGTCTGCTGGACAGCTCACCCTCATGCCCCATATACAGCCTCAGTTCTGAATACTGCGTCCGAAACTCCTGAGGTCCAGCGTGGATCTTCCACAGTCATCTATTTTCTTACGCACACCCATGTACAGGATGAAGCCGAGGCGGCGGAGGGTGACTTTAAGGCCCTCGATCGTATGCGGAATATGGCCATGCAAGCCCGCGCTGCCACGGACACCTCGGTCGCCACATTGCAAGCCTACTACGCCCAAGTGAACCGTCTTCACGGCATCTTTCCCATCAATACCGACCATATCCGCATCGCCTTTACCTGGCACGACGCCTTTCGCCCCACCAAGAAGTCTACAGAAATGGACTTGAACTTTGAGCGGGCAGGCGTACTTTTTAACATTGGGGCCGTCAAGAGCAACATTGCTGCATCGGCAGACCGGACGTCGGCCGAGGGAATGAAACTTGCTTGTCGGGAATTTCAGTTGGCGGCCGGCCTATTCAAGCACTTGCGCTGTCACGTAGTCTCGCAGCTGAATTGTAGCCTACCGTCGGAGCTCACCGAGGAGGGCTTGCTCATGGTGGAGAACATCATGTTGGCACAGGTACGCGTGGTAAAAGGAGCGAAGGAAAATGAAAATCAAGGAAAAGTTTCTTGTAGTTTTTGTCGCTTTCCCTACACCTTTCTTCTGTCCTTCCACAAACTTCTCAGGCCCAAGCCTGTTTTTACGAGAAGTCCGTGGTTGACCACCGGAGCGCCAGCTCAAGCATGAAAGCCTCTGTCGTGGCCCGGCTCGCAGCGCAGGCAGCTGAGATGTACAGCACTGCTCTCAAATACGGGGAGTCTCCAGCCATGGAGGCCGCAGGGCTTGACCGCTCCTGGCCTATTACGCTCCGTTTCCAGCAAGACTTTTACTCGGCCGCAGCGCAGTACTACCAGGCGGAGGCCTGCAAGGAGGCGGCCCAGGAAAATGGCAGTGGCTACGGAGAGGAGGTGACCCGTTTACGAATGGCTCAGACCTACATAGAAGTGGTGCTGGAGACCGCGGCAAGGCAACGCATGGGTCCTGCCATTATTGGGAAAGCCGAACTGTTGAAGGCCAAGATTGTGAAGAACAAAGCAGTGGCAGAGAAGGACAACAGTACCATCTATTTGGAAACGGTGCCAACCGAGGCCTCACTCAAGCCTGTCAATAAGACATGTATGGTTAAGGTCGTTGAGCCCGAGCCCTCTTTCCCATCCACTGGAACCTTGCAGCCCACCCTCTTTGCCAATATCCTTCCCAAGGCCATCAAGGAGGCGTTGACCGAGGCCGAGAAGCAGGTTGTTGCCCTAGCTGCAGCAATGGAGCAGGAGGGTCGGGAAGCATCCAAGGGGGCACGACAACAGCTGGCGAGAGCGGGTCTGCCTGGGTCCCTTCAAGCACACGAAAGCCCCGCCGGCCTCCCGGAGGCAACCTGGAAAAAGCTCCAGGGCCTGAGGGAGAAGCAGTCCACAGTGTCCGACCTCCAGGCTCAGGCAGAGGAATTGCAAAAAACATCTGGACAAGTTGCGGGGACATTTTTGAAGATTGAGAAGATGCTTAAGGAGGAGGAAGTGAGGGAGATCGCTTTTCGTGCTAAGTACGGCCCAGCCTGGGATAAGTTTGGGGAGCCATCCGCACAGCTCATGGCTACTACATGGAAGGAGGTCTCACATTTCCAGACTCTCTTTGCGGCTGCCCAGAGGAGCGACGATTACCTCATCTCGCGCCTGCGCTCTCCTGAACTTGATGGCCTGAGTGCACTGCTGGATGAATCGCGTCGGAATTTGGATGCACGTTTGGCXXXXXXCCAGCCGTCGTCGGAATCGTCGCCCGTGCTGGTGGACTCATCAAATCTCTCGGCGGCCATGCTACGGCTGGCCCGGTTACTGGACGAGCGAGACGCCCTTCTGGTGCGGATTAAGGCGGAGCCTGATGCAATGTATGTGCGGTTAAAGGGACGGCTGTTAGCCGGCGAGCTGGTAAACGAGGCCGTGCCTCCGGCGATGGCCTCTGTGGGGGAGCTCAAGAGTGCATTGGCGGCGTCGATTGCAGGACAAGGGCCCTTAGTGGAGGAGATTTTGAGTGAGAACGAGCGATTTCAAGCAGGGCGGAAGACCGACCCAGCGTTGTTAGAAAGAGATGCACTTGTGCAGCAGCTAGAGACGGGGGTGTTGCAGTGCCACGAGCTTCATGCCCAGTTGGCGGAAGGTCGGGAATTTTATGCTTCGGTAAGCAAGCGAATACAGCAGCTATTGCTGGTGACAGAAGATCAGCTTTACACCCAGGATATCCAACGTCGTGATTTTGAGGTGGAGCTCGGGCAGTCCGCCAATCGGAAGAAGCAAGAAAGTGATGATCAAGATGTGGCCAGGAAGCTTTTCGAGCAGCTCAACGTCCAGCAGCAGCAAGAAGGGGAAGGTGCCGCCACAACCGGAGACGCGCCTCATTTTGTGCCCCAGCCGCCATTTGGTGGTGGTGGACCGTCTCCGTCTGTCCCTCCTCCCCCACCTGCCATTAAGCCTCTTCCTCCTCCCCCCCATTCGCTCTCCACCCCAACTCCGGGGCCACCCCCTTCCTACGAGCAAGCTCAGGCTTTGCCGATCGCGCCTGCCTACACGGAGCAGGTCAAAATATCCCAGTTGGTTCAAATGGGTTTTCCAAAGGCACAAGCTCAGCATGCGCTGGTACAGGAGAGAGGGGATTTACAGGCGGCTCTCAATGTATTGCTCTCGGGCTGA
